# What information and the extent of information research participants need in informed consent forms: a multi-country survey

**DOI:** 10.1186/s12910-018-0318-x

**Published:** 2018-09-15

**Authors:** Juntra Karbwang, Nut Koonrungsesomboon, Cristina E. Torres, Edlyn B. Jimenez, Gurpreet Kaur, Roli Mathur, Eti N. Sholikhah, Chandanie Wanigatunge, Chih-Shung Wong, Kwanchanok Yimtae, Murnilina Abdul Malek, Liyana Ahamad Fouzi, Aisyah Ali, Beng Z. Chan, Madawa Chandratilake, Shoen C. Chiew, Melvyn Y. C. Chin, Manori Gamage, Irene Gitek, Mohammad Hakimi, Narwani Hussin, Mohd F. A. Jamil, Pavithra Janarsan, Madarina Julia, Suman Kanungo, Panduka Karunanayake, Sattian Kollanthavelu, Kian K. Kong, Bing-Ling Kueh, Ragini Kulkarni, Paul P. Kumaran, Ranjith Kumarasiri, Wei H. Lim, Xin J. Lim, Fatihah Mahmud, Jacinto B. V. Mantaring, Siti M. Md Ali, Nurain Mohd Noor, Kopalasuntharam Muhunthan, Elanngovan Nagandran, Maisarah Noor, Kim H. Ooi, Jebananthy A. Pradeepan, Ahmad H. Sadewa, Nilakshi Samaranayake, Shalini Sri Ranganathan, Wasanthi Subasingha, Sivasangari Subramaniam, Nadirah Sulaiman, Ju F. Tay, Leh H. Teng, Mei M. Tew, Thipaporn Tharavanij, Peter S. K. Tok, Jayanie Weeratna, Tri Wibawa, Renu Wickremasinghe, Phanthipha Wongwai, Subhash Yadav, FERCAP Multi-Country Research Team

**Affiliations:** 1 Department of Clinical Product Development, Institute of Tropical Medicine, Nagasaki University, 1-12-4 Sakamoto, Nagasaki, 852-8523 Japan; 2 Department of Pharmacology, Faculty of Medicine, Chiang Mai University, 110 Muang Chiang Mai, Chiang Mai, 50200 Thailand; 3 Forum for Ethical Review Committees in the Asian and Western Pacific region, WHO-TDR Clinical Coordination and Training Center, Thammasat University, Pathum Thani, Thailand; 4 National Institutes of Health, University of the Philippines Manila, Manila, Philippines; 5 Selangor State Health Department, Ministry of Health, Putrajaya, Malaysia; 6 ICMR Bioethics Unit, National Centre for Disease Informatics and Research, Bangalore, India; 7 Department of Pharmacology and Therapy, Faculty of Medicine, Universitas Gadjah Mada, Yogyakarta, Indonesia; 8 Forum for Ethics Review Committees in Sri Lanka and Faculty of Medical Sciences, University of Sri Jayewardanepura, Nugegoda, Sri Lanka; 9 Department of Anesthesiology, Cathay General Hospital, Taipei, Taiwan; 10 Academic Clinical Research Office, Faculty of Medicine, Khon Kaen University, Khon Kaen, Thailand; 11 Kuala Lumpur Hospital, Kuala Lumpur, Malaysia; 12 Sultanah Nur Zahirah Hospital, Kuala Terengganu, Terengganu Malaysia; 13 Sultan Ismail Hospital, Johor Bahru, Johor Malaysia; 14 Melaka Hospital, Melaka, Malaysia; 15 Faculty of Medicine, University of Kelaniya, Ragama, Sri Lanka; 16 Seri Manjung Hospital, Seri Manjung, Perak Malaysia; 17 Sungai Buloh Hospital, Sungai Buloh, Selangor Malaysia; 18 Faculty of Medical Sciences, University of Sri Jayewardenepura, Nugegoda, Sri Lanka; 19 Sarawak General Hospital, Kuching, Malaysia; 20 Medical and Health Research Ethics Committee, Faculty of Medicine Universitas Gadjah Mada, Dr. Sardjito General Hospital, Yogyakarta, Indonesia; 21 Taiping Hospital, Taiping, Malaysia; 22 Seberang Jaya Hospital, Perai, Pulau Pinang Malaysia; 23 Raja Perempuan Zainab II Hospital, Kota Bharu, Malaysia; 24 Department of Pediatric, Faculty of Medicine Universitas Gadjah Mada, Dr. Sardjito General Hospital, Yogyakarta, Indonesia; 25 Division of Epidemiology, National Institute of Cholera & Enteric Diseases, Kolkata, India; 26 Faculty of Medicine, University of Colombo, Colombo, Sri Lanka; 27 Ampang Hospital, Ampang, Malaysia; 28 Duchess of Kent Hospital, Sandakan, Malaysia; 29 Likas Hospital, Kota Kinabalu, Sabah Malaysia; 30 Department of Operational Research, National Institute for Research in Reproductive Health, Mumbai, India; 31 National Institute for Research in Tuberculosis, Chennai, India; 32 Faculty of Medicine, University of Peradeniya, Peradeniya, Sri Lanka; 33 Sibu Hospital, Sibu, Sarawak Malaysia; 34 Raja Permaisuri Bainun Hospital, Ipoh, Malaysia; 35 Tengku Ampuan Afzan Hospital, Kuantan, Malaysia; 36 University of the Philippines Manila Research Ethics Board, Manila, Philippines; 37 Sultanah Bahiyah Hospital, Alor Setar, Kedah Malaysia; 38 Putrajaya Hospital, Putrajaya, Malaysia; 39 Faculty of Medicine, University of Jaffna, Jaffna, Sri Lanka; 40 Tengku Ampuan Rahimah Hospital, Klang, Malaysia; 41 Tuanku Jaáfar Hospital, Seremban, Malaysia; 42 Tuanku Fauziah Hospital, Kangar, Perlis Malaysia; 43 Department of Biochemistry, Faculty of Medicine, Universitas Gadjah Mada, Yogyakarta, Indonesia; 44 Pulau Pinang Hospital, George Town, Malaysia; 45 Queen Elizabeth I Hospital, Kota Kinabalu, Sabah Malaysia; 46 Selayang Hospital, Shah Alam, Malaysia; 47 Miri Hospital, Miri, Sarawak Malaysia; 48 Sultan Abdul Halim Hospital, Sungai Petani, Kedah Malaysia; 49 Endocrinology and Metabolism Unit, Department of Medicine, Faculty of Medicine, Thammasat University, Pathum Thani, Thailand; 50 Sultanah Aminah Hospital,, Johor Bharu, Malaysia; 51 Institute of Forensic Medicine and Toxicology, Colombo, Sri Lanka; 52 Department of Microbiology, Faculty of Medicine, Universitas Gadjah Mada, Yogyakarta, Indonesia; 53 Department of Opthalmology, Faculty of Medicine, Khon Kaen University, Khon Kaen, Thailand; 54 Department of Endocrinology, Sanjay Gandhi Post Graduate Institute of Medical Sciences (SGPGI), Lucknow, Uttar Pradesh India

**Keywords:** Consent forms, Informed consent, Disclosure, Information, Ethics, Research subjects

## Abstract

**Background:**

The use of lengthy, detailed, and complex informed consent forms (ICFs) is of paramount concern in biomedical research as it may not truly promote the rights and interests of research participants. The extent of information in ICFs has been the subject of debates for decades; however, no clear guidance is given. Thus, the objective of this study was to determine the perspectives of research participants about the type and extent of information they need when they are invited to participate in biomedical research.

**Methods:**

This multi-center, cross-sectional, descriptive survey was conducted at 54 study sites in seven Asia-Pacific countries. A modified Likert-scale questionnaire was used to determine the importance of each element in the ICF among research participants of a biomedical study, with an anchored rating scale from 1 (not important) to 5 (very important).

**Results:**

Of the 2484 questionnaires distributed, 2113 (85.1%) were returned. The majority of respondents considered most elements required in the ICF to be ‘moderately important’ to ‘very important’ for their decision making (mean score, ranging from 3.58 to 4.47). Major foreseeable risk, direct benefit, and common adverse effects of the intervention were considered to be of most concerned elements in the ICF (mean score = 4.47, 4.47, and 4.45, respectively).

**Conclusions:**

Research participants would like to be informed of the ICF elements required by ethical guidelines and regulations; however, the importance of each element varied, e.g., risk and benefit associated with research participants were considered to be more important than the general nature or technical details of research. Using a participant-oriented approach by providing more details of the participant-interested elements while avoiding unnecessarily lengthy details of other less important elements would enhance the quality of the ICF.

**Electronic supplementary material:**

The online version of this article (10.1186/s12910-018-0318-x) contains supplementary material, which is available to authorized users.

## Background

An informed consent form (ICF) is mandatory and essential in most studies involving human subjects as it is a primary vehicle for disclosure of information and documentation of consent [1, 2]. An observation of the current research practice reveals that ICFs continue to increase in length and complexity in an attempt to comply with regulatory requirements [3–6], which increasingly require more and more elements to address past and present unethical practice [7, 8]. Lengthy and complex ICFs decrease the ability of potential participants to comprehend the ICF content and exercise their autonomy in decision making to participate in a study [9]. ICFs have been gradually turned into legal documents for the protection of researchers and sponsors rather than documents with relevant information for decision making of research participants [10].

In an attempt to make ICFs comprehensible, the extent of information disclosure has been the subject of debates [11–13]. Lengthy ICFs with full disclosure of everything may obscure the important and relevant information for decision making whether to participate in a study [14]. Exhaustive disclosure of detailed information of every single aspect related to the study may overwhelm potential research participants with too excessive information [15]. Based on a systematic review on the desired information by potential participants of biomedical research, there is limited empirical evidence on this subject [16]. Generally, the type and extent of information considered as adequate and relevant for a person to make a decision are subjective and difficult to define. In addition, some information perceived as relevant and important by some research participants, with respect to their cultural context, may be absent in even a lengthy ICF as it is not required by applicable laws and regulations [13]. To address these issues, empirical data related to the content and extent of information that research participants require for their decision making are needed. The objective of this study was to determine the perspectives of research participants about the information they need for their decision making when they are invited to participate in biomedical research.

## Methods

### Study design and settings

This multi-center, cross-sectional, descriptive survey was conducted by the Forum for Ethical Review Committees in the Asian and Western Pacific region (FERCAP) Multi-Country Research Team in 7 FERCAP-member countries, i.e., India, Indonesia, Malaysia, Philippines, Sri Lanka, Taiwan, and Thailand. The duration of this study was three months, from June 1 to August 31, 2017, with a two-week extension in some countries where a sample size was not met within the three-month period.

### Study material

An anonymous, paper-based, structured and self-administered questionnaire was developed and reviewed by a group of FERCAP professionals with expertise in research surveys, biomedical research, research ethics, and informed consent. Survey items were developed based on the essential elements required by three major ethical guidelines and regulations, i.e., Declaration of Helsinki [1], the International Conference on Harmonization (ICH) for Good Clinical Practice (GCP) [2], and US Code of Federal Regulations [17]. A content validity test was conducted to establish that individual items were relevant to the construct being measured and that key items had not been omitted in the questionnaire [18]. The questionnaire asked participants to indicate how important each item was by giving a rating from 1 to 5 using a modified Likert scale [19]: 1 = not important, 2 = slightly important, 3 = moderately important, 4 = fairly important, and 5 = very important. There were open-ended questions in the questionnaire where the participants could suggest any additional elements or information that they would like to receive. Demographic data (age, sex, educational level, nationality, and occupation) and the participants’ preference in page length were included in the questionnaire. The think-aloud technique was used to assess how respondents interpreted each item and response anchor [20]. The questionnaire was then finalized and translated into the local language for use in each participating country. The questionnaires in local languages were back-translated into English by independent individuals who are fluent in both local and English languages and checked against the original questionnaire. The translated questionnaire was piloted in a small group of individuals who were members of the target population within each respective country.

### Study population and sample size determination

This study enrolled individuals who were participating in ongoing biomedical research – a research study relating to biology and medicine for healthcare purposes – at various participating centers (clinical research units or comparable settings) in 7 countries. Individuals who refused to answer the questionnaire for any reason or had communication difficulties due to language problems or cognitive disabilities were excluded.

The sample size for this study was meant to yield a representative sample under the assumption that the quantity of interest is measured by a Likert scale. Following the formula described in Park & Jung (2009) [21], a sample size of at least 231 would be adequate when a 5-point scale was used for each Likert-item (k = 5), with a coefficient of variation of a population (C) = 0.5, a relative tolerable error (D) = 5%, and a pairwise correlation coefficient (ρ) = 0.5. The present study was initially planned to enrol, at least, 300 participants in each country based on an approximate estimate of 20% of missing data due to some participants who might skip certain questions or items that they were not comfortable with.

### Study procedure and data collection

Data were collected through an anonymous, self-administered, structured, paper-based questionnaire. No written consent was required for this survey study as an individual participant’s voluntary completion of the questionnaire was presumed as consent. Site investigators collaborated with research staff at clinical research units or comparable settings and informed research participants in biomedical research about this ICF study. Instructions were provided by research staff to potential participants that they could refuse to answer the questionnaire for any reason; they could skip any question that they were unwilling to answer; and they would not be treated differently because of the responses they gave in the questionnaire. When participants agreed to take part in this survey, research staff gave them the questionnaire. The participants could complete the questionnaire at any time and returned it to the collection box located at participating centers.

### Ethical considerations

The study was conducted in compliance with the Declaration of Helsinki 2013. The study protocol and related documents obtained ethical approval from local ethics committees prior to the commencement of the survey in each center. This study was considered ‘minimal-risk’ research since it involved only the use of a questionnaire with no sensitive questions. The information was recorded in an anonymous manner. The participants could skip any question that they did not want to answer. Answering the questionnaire and returning it to the collection box implied the participants’ voluntary consent for the investigators to use their answers to meet the research objective. No separate written consent was required to ensure the participants’ anonymity.

### Statistical analysis

Data from 7 countries were gathered, analyzed, and presented as frequency and percentage, mean and standard deviation (SD), or median and interquartile range (IQR), as appropriate. For the participants’ preference in page length, ‘no limit’ or more than 30 pages was transformed to the value of 30 for analysis. Likert scale responses of each item were analyzed using parametric approaches [22–24]. Differences of variables among countries were done using the one-way analysis of variance (ANOVA), followed by Tukey post hoc test. The association between independent variables (i.e., sex, age, education, occupation, and type of research involved) and the mean score of each item was assessed using multivariable regression analysis. Statistical analyses were performed using SPSS (IBM Corp. Release 2013. IBM SPSS Statistics for Windows, Version 22.0. Armonk, NY: IBM Corp). A *p* value of less than 0.05 was considered to indicate statistical significance.

## Results

This FERCAP Multi-Country ICF study recruited research participants from 54 study sites in 7 countries. Of the 2484 questionnaires distributed, 2113 (85.1%) were returned (Table 1). Demographic data of the respondents are shown in Table 2. The majority of the respondents were female (57.9%), middle-aged adults (aged 43.3 ± 16.2 years, range 15–90 years), had a high-school level of education or lower (64.5%).

**Table 1:** Number of questionnaires distributed and collected by each country

Country	Site (*n*)	Questionnaires distributed (*n*)	Questionnaires collected (*n*)	
India	4	434	410	(94.5%)
Indonesia	1	362	299	(82.6%)
Malaysia	28	508	508	(100.0%)
Philippines	12	508	267	(52.6%)
Sri Lanka	6	335	303	(90.4%)
Taiwan	1	229	229	(100.0%)
Thailand	2	108	97	(89.8%)
	**54**	**2484**	**2113**	**(85.1%)**

**Table 2:** Demographic data of the respondents

Characteristics of the respondents	*n* (%)	
Sex		
Male	884	(42.1%)
Female	1215	(57.9%)
Age		
15–30 years	638	(30.4%)
31–45 years	546	(26.0%)
46–60 years	514	(24.5%)
61–90 years	401	(19.1%)
Educational level		
High school or lower	1331	(64.5%)
Bachelor/diploma degree	525	(25.4%)
Master/doctor degree	208	(10.1%)
Occupation		
Healthcare profession	245	(12.4%)
Non-healthcare profession^†^	1730	(87.6%)
Type of research involved		
Experimental research	1234	(59.8%)
Observational research	830	(40.2%)

^†^Non-healthcare profession, including students, housewives, retirees, and the unemployed

**Fig. 1:**
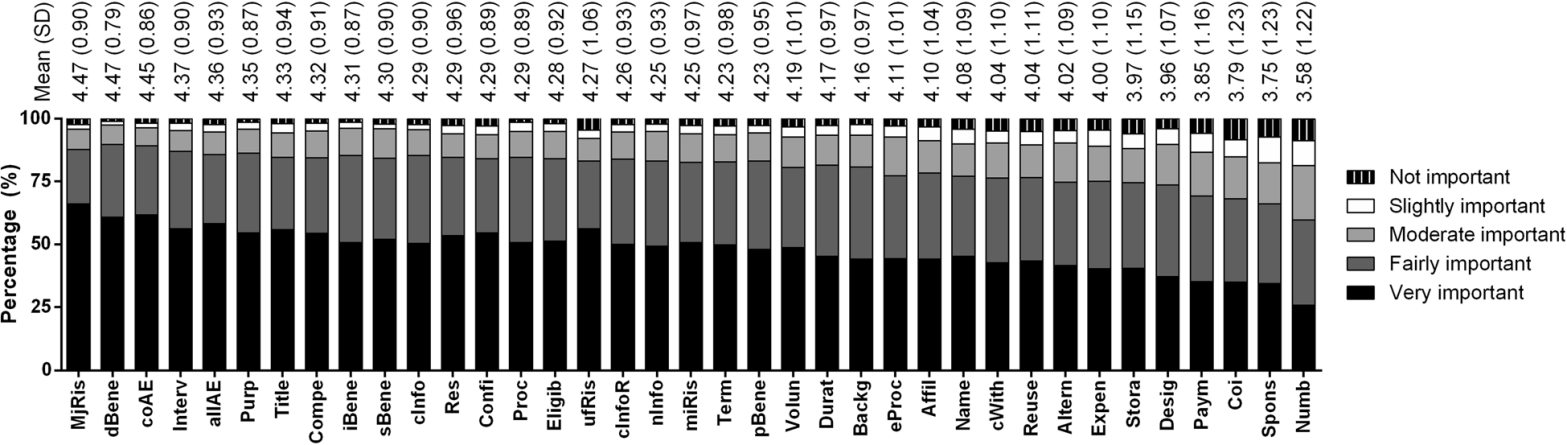
The element and extent of information that research participants wanted to receive

Overall, the respondents wanted to know most elements of the ICF content required (Table 3), with mean scores ranging from 3.58 (about wanting to know the number of participants) to 4.47 (for major foreseeable risk) as shown in Fig. 1. All of the 37 items were rated as ‘moderately important’ or higher among approximately 80% of the respondents to as high as 97.4% of them highly interested in the direct benefit of research (see Additional file 1: Table S1). None of the items were considered ‘slightly important’ or lower in more than one-third of the respondents from any country. Statistically significant differences were found among countries in the mean score of all the 37 items when one-way ANOVA was applied (*p* < 0.001) (data not shown).

**Table 3:** The element and extent of information that research participants wanted to receive

Element	Abbreviation	*n*	Extent of information				
			Mean	SE	SD	Median	IQR
*1. General items*							
1.1 Title of research	Title	(*n* = 2100)	4.33	0.021	0.941	5	(4–5)
1.2 Name of researchers	Name	(*n* = 2091)	4.08	0.024	1.088	4	(4–5)
1.3 Affiliation or organization of researchers	Affil	(*n* = 2083)	4.10	0.023	1.035	4	(4–5)
1.4 Recognition that this is research	Resea	(*n* = 2095)	4.29	0.021	0.963	5	(4–5)
1.5 Contact information regarding the research study	cInfo	(*n* = 2040)	4.29	0.020	0.899	5	(4–5)
1.6 Contact information about the participant’s right	cInfoR	(*n* = 2035)	4.26	0.021	0.932	5	(4–5)
1.7 Source of funds and sponsors	Spons	(*n* = 2082)	3.75	0.027	1.233	4	(3–5)
1.8 Conflict of interest	Coi	(*n* = 2020)	3.79	0.027	1.228	4	(3–5)
*2. Study-specific items*							
2.1 Background and rationale of research	Backg	(*n* = 2084)	4.16	0.021	0.966	4	(4–5)
2.2 Purpose of research	Purp	(*n* = 2093)	4.35	0.019	0.868	5	(4–5)
2.3 Eligibility of the participant	Eligib	(*n* = 2104)	4.28	0.020	0.915	5	(4–5)
2.4 Study design of research	Desig	(*n* = 2078)	3.96	0.023	1.071	4	(3–5)
2.5 Interventions under investigation	Interv	(*n* = 2080)	4.37	0.020	0.895	5	(4–5)
2.6 Common adverse effects of the intervention	coAE	(*n* = 2082)	4.45	0.019	0.857	5	(4–5)
2.7 All possible adverse effects of the intervention	allAE	(*n* = 2086)	4.36	0.020	0.933	5	(4–5)
2.8 Other options or alternative treatments	Altern	(*n* = 2088)	4.02	0.024	1.093	4	(3–5)
2.9 Duration of the participant’s participation	Durat	(*n* = 2069)	4.17	0.021	0.971	4	(4–5)
2.10 Schedule and procedure	Proc	(*n* = 2089)	4.29	0.020	0.894	5	(4–5)
2.11 Identification of any experimental procedures	eProc	(*n* = 2075)	4.11	0.022	1.013	4	(4–5)
2.12 Number of participants involved	Numb	(*n* = 2092)	3.58	0.027	1.218	4	(3–5)
2.13 Criteria for termination	Term	(*n* = 2045)	4.23	0.022	0.975	4	(4–5)
*3. Items related to the subject’s right*							
3.1 Voluntary participation	Volun	(*n* = 2093)	4.19	0.022	1.014	4	(4–5)
3.2 Consequence of withdrawal	cWith	(*n* = 2039)	4.04	0.024	1.097	4	(4–5)
3.3 Right to receive new information	nInfo	(*n* = 2048)	4.25	0.020	0.927	4	(4–5)
*4. Items related to risk-benefit*							
4.1 Major foreseeable risk	mjRis	(*n* = 2077)	4.47	0.020	0.902	5	(4–5)
4.2 Minor foreseeable risk	miRis	(*n* = 2077)	4.25	0.021	0.968	5	(4–5)
4.3 Possibly unforeseeable risk	ufRis	(*n* = 2043)	4.27	0.024	1.064	5	(4–5)
4.4 Direct health benefit	dBene	(*n* = 2088)	4.47	0.017	0.793	5	(4–5)
4.5 Indirect benefit	iBene	(*n* = 2096)	4.31	0.019	0.865	5	(4–5)
4.6 Societal benefit	sBene	(*n* = 2049)	4.30	0.020	0.901	5	(4–5)
4.7 Post-trial benefit or provision	pBene	(*n* = 2048)	4.23	0.021	0.950	4	(4–5)
*5. Items related to data and sample storage*							
5.1 Confidentiality and the limit of confidentiality	Confi	(*n* = 2048)	4.29	0.022	0.984	5	(4–5)
5.2 Storage of human material	Stora	(*n* = 2041)	3.97	0.025	1.148	4	(3–5)
5.3 Reuse of human material	Reuse	(*n* = 2043)	4.04	0.025	1.113	4	(4–5)
*6. Items related to monetary issues*							
6.1 Payment and/or remuneration	Paym	(*n* = 2040)	3.85	0.026	1.156	4	(3–5)
6.2 Anticipated expense	Expen	(*n* = 2025)	4.00	0.024	1.099	4	(4–5)
6.3 Compensation for injury	Compe	(*n* = 2038)	4.32	0.020	0.912	5	(4–5)

*IQR* interquartile range, *SD* standard deviation, *SE* standard error of the mean

Major foreseeable risk, direct benefit, and common adverse effects of the intervention were considered to be of most concern among the respondents (with mean scores of 4.47, 4.47, and 4.45, respectively) as shown in Fig. 1. In contrast, items about payment and/or remuneration, conflict of interest, the source of funds and sponsors, and the number of participants involved were considered to be of relatively less concern (with mean scores of 3.85, 3.79, 3.75, and 3.58, respectively). Nevertheless, there was slight variation in the items that were of most and of least concern among research participants in different countries (Table 4).

**Table 4:**
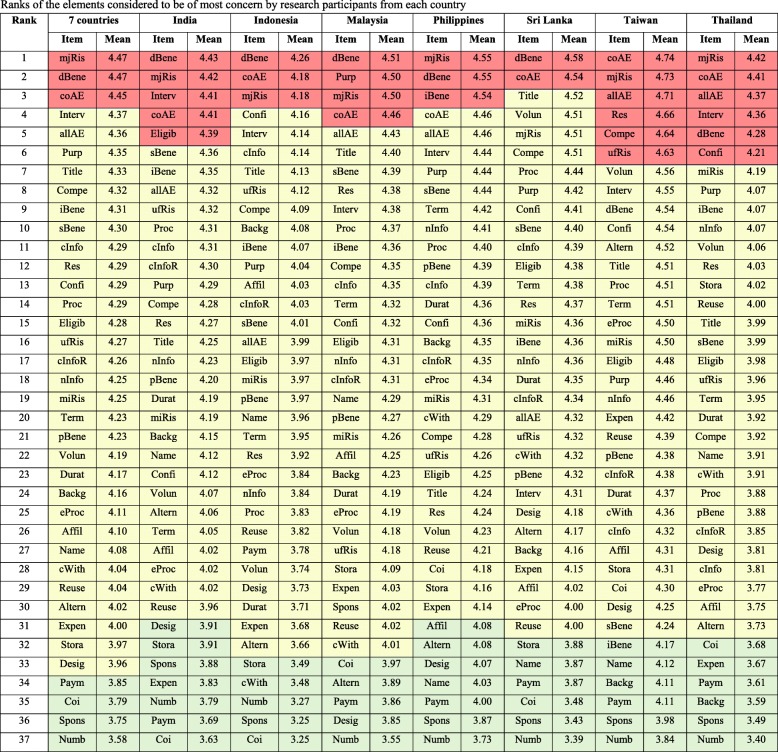
Ranks of the elements considered to be of most concern by research participants from each country

The values that are more than ‘mean + 1 SD’ (considered to be of most concern) is in RED columns; those within ‘mean ± 1 SD’ is in YELLOW columns; and those less than ‘mean – 1 SD’ (considered to be of least concern) is in GREEN columns

Demographic characteristics were found to be associated with the scores in several items. Higher scores in the desire to receive detailed information were associated with female gender (in 34 out of the 37 items), healthcare profession (in 24 out of the 37 items), younger age (in 11 out of the 37 items), and higher educational levels (in 10 out of the 37 items) (see Additional file 2: Table S2). Those participating in experimental research wanted more information in 30 out of the 37 items, as compared to those participating in observational research.

The maximum, acceptable number of pages in the ICF that research participants preferred to read was 6.3 ± 6.1 pages (median, 5 pages; IQR, 2–8 pages). However, this value varied among countries. The Taiwanese respondents reported the longest maximum, acceptable number of pages that they would read (12.3 ± 10.8 pages, *n* = 174), followed by the Filipino (7.7 ± 6.5 pages, *n* = 200), Malaysian (7.0 ± 5.7 pages, *n* = 438), Thai (5.3 ± 3.1 pages, *n* = 22), Indian (5.0 ± 3.2 pages, *n* = 378), Indonesian (4.1 ± 3.3 pages, *n* = 242), and Sri Lankan respondents (3.7 ± 3.3 pages, *n* = 213) (see Additional file 3: Table S3). Multivariable analysis identified factors that were associated with the maximum, acceptable number of pages in the ICF, i.e., occupation (healthcare profession vs. non-healthcare profession, *p* < 0.001) and type of research involved (experimental vs. observational, *p* < 0.001). When compared to their counterparts, research participants in the healthcare profession and those participating in the experimental research were more agreeable to reading longer ICFs (8.6 vs. 5.9 pages, *p* < 0.001; 6.8 vs. 5.5 pages, *p* < 0.001, respectively) (see Additional file 4: Figure S1).

There were 58 comments from 56 respondents, suggesting additional information needs. The majority of these comments showed their desire to know about whether to be informed of research results (n = 37) and the location where the research will be conducted (*n* = 8). A few respondents mentioned that they wanted to receive information about legal liability related to research (n = 4), other study sites involved (n = 2), clinical phase of the trial (n = 2), status of the trial, testimony, and approval (n = 2), research budget (n = 2), and related sources of information (n = 1).

## Discussion

This FERCAP Multi-Country ICF study is the largest empirical study on this subject, involving 2113 actual research participants from 54 study sites in 7 countries. It attempted to determine the information that research participants considered to be of importance for their decision making whether to participate in biomedical research. The results indicate that the ICF elements required by ethical guidelines and regulations concur with the information the majority of research participants in 7 Asia-Pacific countries want to know.

The top three items which were of most concern to the respondents in this study were related to the concepts of risks and benefits (i.e., major foreseeable risk, direct benefit, and common adverse effects of the intervention). This finding is consistent with previous numerous studies indicating that research participants regard the risk and benefit associated with their participation to be more important than the general nature or technical details of research [25–27]. Thus, such information should be made a salient feature of an ICF when enrolling potential participants. While the written ICF provided in biomedical research should contain the necessary elements, it should be properly edited and streamlined to ensure concise information to reflect the results of this study. A participant-oriented approach that considers the importance of each element and emphasis on items perceived as more important than others could adequately address participant needs and avoid unnecessarily lengthy details that are of no interest to them [13]. Information should be provided to the extent that it does not detract from what participants want to know and what is needed for a valid consent (i.e., sufficient information, comprehension, and voluntariness) [28]. Information relating to the concepts of risks and benefits, for example, should be described extensively and made salient to potential research participants, while the general nature or technical details of research can be described briefly.

Disclosure requirements based on the elements required in the three major ethical guidelines and regulations – the Declaration of Helsinki [1], ICH GCP [2], and US federal regulations [17] – are generally sufficient to cover all the aspects that most research participants would like to know. However, this study identified additional information that some participants want to be informed about. Information regarding the disclosure of individual results to participants at the end of the study is one of the elements that a sizable number of participants would like to know. This finding is in line with a recent systematic review reporting that several participants wanted to be told about dissemination of study results [16]. This issue has lately been addressed in the revised US Federal Policy for the Protection of Human Subjects, promulgated in January 2017, that requires *“a statement regarding whether clinically relevant research results, including individual research results, will be disclosed to subjects, and if so, under what conditions”* [7]. Nevertheless, it is important to note that disclosure of individual research results (IRRs) might pose psychosocial risks to research participants and their relatives in some settings, especially in genetic-association research [29]. Hence, conditions for disclosure of IRRs should be predefined, e.g., the results that will be conveyed to the participants should be analytically valid, medically important, and actionable, with respect to the participants’ preference [30–32]. In addition, a few respondents raised concerns about legal liability related to research while another wanted to know other study sites involved. When individuals would like to obtain certain additional information that may be relevant to their concerns, investigators may be required to disclose or provide them on a case-by-case basis [28].

The analysis of the respondents’ acceptable page length suggests that an approximately 6-page-long ICF seems to be acceptable to general populations in 7 countries across the Asia-Pacific region. This result is in line with other evidence promoting the use of short and concise ICFs in biomedical research [33]. As shown in a previous empirical study on the preferred length of ICFs, most participants preferred concise, rather than detailed information when they made a decision on trial participation [11]. Another evidence also suggested that a concise ICF is as valid as a detailed, standard ICF to comply with ethical requirements [34]. Although concise forms may not be able to improve participants’ satisfaction with the consent process in all settings, they still have other advantages to the readers as ones are less likely to thoroughly read long forms and wholly absorb extensive information [35]. Recently, there has been a major change in the ethical guidelines and regulations that encourages investigators and sponsors to summarize relevant and important information in a few pages [7, 8]. An ICF used in biomedical research should no longer be an unduly long document, with key information often being hard to find [33]. Concise ICFs with complete information as required by regulations can be developed, for example, using the SIDCER ICF methodology, which has recently been validated and published [36–39]. This methodology requires a thorough understanding of the protocol followed by the summarized information relevant to the interest and concerns of research participants [36]. Visual aids such as summary tables, highlighting keywords and pictographs should be used, when appropriate, to simplify and help participants understand detailed and complex information [36, 40]. Nevertheless, some groups of participants, such as the Taiwanese, might indicate a preference for a relatively longer ICF which contains more detailed and comprehensive information. Supplementary provision of detailed information could be offered to such groups in additional papers (e.g., appendices) or via websites [25, 33].

A closer examination revealed that female participants, healthcare professionals, younger age groups, and those with high educational levels wanted to receive more information about several items when compared to their counterparts. This indicates the different needs of different groups for relevant biomedical or clinical trial information [41]. Furthermore, research participants from different countries showed slight variation in their interests in each element. This is in line with other studies which suggested that information needs may somewhat vary across diverse socioeconomic backgrounds and cultural settings [42–44]. However, there is also a possibility that the difference of variables among countries might not be a genuine difference in ethical views; rather, it might be influenced by response styles across countries or cultural backgrounds [45].

The results of this extensive multi-country survey, involving over 2000 actual research participants at 54 study sites across 7 Asia-Pacific countries, may be considered to be representative of the perspectives of general populations in the Asia-Pacific region. However, this study has a limitation as it lacks data on the perspectives of those asked to provide surrogate consent for others (e.g., parents or other legally acceptable representatives). It is reasonable to assume that the content and extent of information needed among surrogates may be different from what we observed among actual research participants in this study [46, 47]. In addition, different levels of research risk (e.g., low-risk studies with little or no intervention versus high-risk studies with invasive interventions) may result in different needs for trial information among research participants [25, 40]. Further research is required to help tailor ICFs toward more specific types of biomedical research, including biobank research, and population subgroups, such as the study previously done by Casarett et al. for pain research [48].

## Conclusions

In summary, what research participants would like to be informed of mostly concurred with the elements of the ICF content required by the current ethical guidelines and regulations. However, some elements may be more important than others and such information should be made salient to research participants. The study results provide important insights to better address the challenges of determining the extent of information in ICFs that is considered to be important and adequate from research participants’ perspectives.

## Additional files

Additional file 1: **Table S1.** The proportions of the respondents who wanted to know each element. (DOCX 20 kb)

Additional file 2: **Table S2.** Associations between the respondents’ characteristics and their desire to know each element of the ICF content. (DOCX 28 kb)

Additional file 3: **Table S3.** The maximum, acceptable number of pages in the informed consent form and its comparisons among countries. (DOCX 16 kb)

Additional file 4: **Figure S1.** Differences in the acceptable page length among respondents with different genders, educational levels, occupations, and types of research involved. (TIF 512 kb)
