# Supplementary material for: What information and the extent of information research participants need in informed consent forms: a multi-country survey
Source: BMC Med Ethics. 2018 Sep 15;19:79. doi: 10.1186/s12910-018-0318-x (PMC6139128; doi:10.1186/s12910-018-0318-x)
Supplement: Supplementary file 1 — Table S1. The proportions of the respondents who wanted to know each element. (DOCX 20 kb) [file 12910_2018_318_MOESM1_ESM.docx]

**Table S2** The proportions of the respondents who wanted to know each element

| **Element** | **7 countries** | | **India** | | **Indonesia** | | **Malaysia** | | **Philippines** | | **Sri Lanka** | | **Taiwan** | | **Thailand** | |
| --- | --- | --- | --- | --- | --- | --- | --- | --- | --- | --- | --- | --- | --- | --- | --- | --- |
| Title | 1979/2100 | (94.2%) | 375/410 | (91.5%) | 285/291 | (97.9%) | 474/507 | (93.5%) | 252/266 | (94.7%) | 278/301 | (92.4%) | 225/228 | (98.7%) | 90/97 | (92.8%) |
| Name | 1880/2091 | (89.9%) | 361/410 | (88.0%) | 269/286 | (94.1%) | 471/505 | (93.3%) | 239/265 | (90.2%) | 233/299 | (77.9%) | 220/229 | (96.1%) | 87/97 | (89.7%) |
| Affil | 1902/2083 | (91.3%) | 354/404 | (87.6%) | 273/284 | (96.1%) | 475/503 | (94.4%) | 240/267 | (89.9%) | 251/299 | (83.9%) | 225/229 | (98.3%) | 84/97 | (86.6%) |
| Spons | 1716/2082 | (82.4%) | 331/400 | (82.8%) | 211/291 | (72.5%) | 450/501 | (89.8%) | 230/266 | (86.5%) | 202/299 | (67.6%) | 216/229 | (94.3%) | 76/96 | (79.2%) |
| Resea | 1969/2095 | (94.0%) | 380/406 | (93.6%) | 265/293 | (90.4%) | 479/504 | (95.0%) | 250/266 | (94.0%) | 274/300 | (91.3%) | 227/229 | (99.1%) | 94/97 | (96.9%) |
| Eligib | 1997/2104 | (94.9%) | 392/407 | (96.3%) | 281/298 | (94.3%) | 478/506 | (94.5%) | 253/267 | (94.8%) | 276/300 | (92.0%) | 227/229 | (99.1%) | 90/97 | (92.8%) |
| Numb | 1705/2092 | (81.5%) | 332/403 | (82.4%) | 225/298 | (75.7%) | 411/502 | (81.9%) | 230/265 | (86.8%) | 217/299 | (72.6%) | 210/228 | (92.1%) | 80/97 | (82.5%) |
| Volun | 1939/2093 | (92.6%) | 364/409 | (89.0%) | 263/292 | (90.1%) | 463/503 | (92.0%) | 251/266 | (94.4%) | 280/298 | (94.0%) | 225/228 | (98.7%) | 93/97 | (95.9%) |
| Altern | 1884/2088 | (90.2%) | 373/410 | (91.0%) | 255/291 | (87.6%) | 438/504 | (86.9%) | 248/261 | (95.0%) | 256/297 | (86.2%) | 225/228 | (98.7%) | 89/97 | (91.8%) |
| Backg | 1946/2084 | (93.4%) | 368/406 | (90.6%) | 283/292 | (96.9%) | 473/507 | (93.3%) | 251/255 | (98.4%) | 268/299 | (89.6%) | 221/228 | (96.9%) | 82/97 | (84.5%) |
| Interv | 1980/2080 | (95.2%) | 386/406 | (95.1%) | 280/292 | (95.9%) | 477/504 | (94.6%) | 251/256 | (98.0%) | 263/296 | (88.9%) | 227/229 | (99.1%) | 96/97 | (99.0%) |
| coAE | 2003/2082 | (96.2%) | 379/401 | (94.5%) | 281/292 | (96.2%) | 482/506 | (95.3%) | 258/261 | (98.9%) | 280/296 | (94.6%) | 227/229 | (99.1%) | 96/97 | (99.0%) |
| allAE | 1976/2086 | (94.7%) | 377/406 | (92.9%) | 270/292 | (92.5%) | 480/504 | (95.2%) | 258/262 | (98.5%) | 269/296 | (90.9%) | 226/229 | (98.7%) | 96/97 | (99.0%) |
| Purp | 2008/2093 | (95.9%) | 385/410 | (93.9%) | 278/291 | (95.5%) | 485/507 | (95.7%) | 258/263 | (98.1%) | 281/297 | (94.6%) | 226/228 | (99.1%) | 95/97 | (97.9%) |
| Desig | 1863/2078 | (89.7%) | 349/403 | (86.6%) | 257/290 | (88.6%) | 438/500 | (87.6%) | 245/261 | (93.9%) | 260/298 | (87.2%) | 223/229 | (97.4%) | 91/97 | (93.8%) |
| Durat | 1932/2069 | (93.4%) | 372/404 | (92.1%) | 256/287 | (89.2%) | 465/501 | (92.8%) | 252/258 | (97.7%) | 271/293 | (92.5%) | 226/229 | (98.7%) | 90/97 | (92.8%) |
| Proc | 1982/2089 | (94.9%) | 388/409 | (94.9%) | 264/292 | (90.4%) | 482/503 | (95.8%) | 256/262 | (97.7%) | 276/297 | (92.9%) | 227/229 | (99.1%) | 89/97 | (91.8%) |
| eProc | 1922/2075 | (92.6%) | 361/401 | (90.0%) | 265/290 | (91.4%) | 468/502 | (93.2%) | 259/263 | (98.5%) | 253/294 | (86.1%) | 227/229 | (99.1%) | 89/96 | (92.7%) |
| mjRis | 1987/2077 | (95.7%) | 381/403 | (94.5%) | 278/292 | (95.2%) | 480/501 | (95.8%) | 255/260 | (98.1%) | 274/296 | (92.6%) | 225/229 | (98.3%) | 94/96 | (97.9%) |
| miRis | 1953/2077 | (94.0%) | 377/405 | (93.1%) | 268/292 | (91.8%) | 466/501 | (93.0%) | 252/259 | (97.3%) | 271/295 | (91.9%) | 223/228 | (97.8%) | 96/97 | (99.0%) |
| ufRis | 1879/2043 | (92.0%) | 379/405 | (93.6%) | 274/292 | (93.8%) | 427/481 | (88.8%) | 237/252 | (94.0%) | 250/288 | (86.8%) | 225/228 | (98.7%) | 87/97 | (89.7%) |
| dBene | 2033/2088 | (97.4%) | 393/407 | (96.6%) | 286/292 | (97.9%) | 487/503 | (96.8%) | 262/265 | (98.9%) | 283/295 | (95.9%) | 225/229 | (98.3%) | 97/97 | (100%) |
| iBene | 2016/2096 | (96.2%) | 394/407 | (96.8%) | 283/293 | (96.6%) | 482/506 | (95.3%) | 262/265 | (98.9%) | 278/300 | (92.7%) | 222/228 | (97.4%) | 95/97 | (97.9%) |
| sBene | 1967/2049 | (96.0%) | 393/409 | (96.1%) | 283/297 | (95.3%) | 483/504 | (95.8%) | 262/265 | (98.9%) | 231/248 | (93.1%) | 222/229 | (96.9%) | 93/97 | (95.9%) |
| cWith | 1843/2039 | (90.4%) | 356/405 | (87.9%) | 246/299 | (82.3%) | 450/503 | (89.5%) | 254/262 | (96.9%) | 218/244 | (89.3%) | 226/229 | (98.7%) | 93/97 | (95.9%) |
| nInfo | 1943/2048 | (94.9%) | 382/407 | (93.9%) | 272/298 | (91.3%) | 476/503 | (94.6%) | 263/265 | (99.2%) | 229/249 | (92.0%) | 226/229 | (98.7%) | 95/97 | (97.9%) |
| Term | 1915/2045 | (93.6%) | 359/405 | (88.6%) | 280/299 | (93.6%) | 471/503 | (93.6%) | 260/265 | (98.1%) | 225/247 | (91.1%) | 226/229 | (98.7%) | 94/97 | (96.9%) |
| pBene | 1931/2048 | (94.3%) | 385/407 | (94.6%) | 283/299 | (94.6%) | 467/506 | (92.3%) | 258/263 | (98.1%) | 221/247 | (89.5%) | 227/229 | (99.1%) | 90/97 | (92.8%) |
| Confi | 1916/2048 | (93.6%) | 373/409 | (91.2%) | 278/299 | (93.0%) | 467/506 | (92.3%) | 257/263 | (97.7%) | 221/246 | (89.8%) | 225/228 | (98.7%) | 95/97 | (97.9%) |
| Stora | 1798/2041 | (88.1%) | 348/407 | (85.5%) | 239/292 | (81.8%) | 449/507 | (88.6%) | 250/262 | (95.4%) | 195/247 | (78.9%) | 221/229 | (96.5%) | 96/97 | (99.0%) |
| Reuse | 1829/2043 | (89.5%) | 356/406 | (87.7%) | 258/298 | (86.6%) | 449/505 | (88.9%) | 253/263 | (96.2%) | 194/245 | (79.2%) | 224/229 | (97.8%) | 95/97 | (97.9%) |
| Paym | 1768/2040 | (86.7%) | 339/408 | (83.1%) | 255/298 | (85.6%) | 428/505 | (84.8%) | 246/260 | (94.6%) | 193/243 | (79.4%) | 222/229 | (96.9%) | 85/97 | (87.6%) |
| Expen | 1803/2025 | (89.0%) | 349/407 | (85.7%) | 253/298 | (84.9%) | 451/506 | (89.1%) | 247/259 | (95.4%) | 205/245 | (83.7%) | 227/229 | (99.1%) | 71/81 | (87.7%) |
| Compe | 1938/2038 | (95.1%) | 389/409 | (95.1%) | 275/291 | (94.5%) | 476/507 | (93.9%) | 252/261 | (96.6%) | 229/245 | (93.5%) | 226/228 | (99.1%) | 91/97 | (93.8%) |
| cInfo | 1952/2040 | (95.7%) | 387/409 | (94.6%) | 290/297 | (97.6%) | 479/505 | (94.9%) | 260/263 | (98.9%) | 220/241 | (91.3%) | 224/228 | (98.2%) | 92/97 | (94.8%) |
| cInfoR | 1927/2035 | (94.7%) | 379/408 | (92.9%) | 280/295 | (94.9%) | 473/504 | (93.8%) | 259/263 | (98.5%) | 218/240 | (90.8%) | 224/228 | (98.2%) | 94/97 | (96.9%) |
| Coi | 1711/2020 | (84.7%) | 321/406 | (79.1%) | 211/286 | (73.8%) | 447/504 | (88.7%) | 250/261 | (95.8%) | 168/238 | (70.6%) | 224/228 | (98.2%) | 90/97 | (92.8%) |

The 5-item Likert scale was transformed into the binary outcome: ‘1’ or ‘2’ = did not want to know; ‘3’ or ‘4’ or ‘5’ = wanted to know. The data are presented as ‘proportion (percentage)’.
