# Supplementary material for: What information and the extent of information research participants need in informed consent forms: a multi-country survey
Source: BMC Med Ethics. 2018 Sep 15;19:79. doi: 10.1186/s12910-018-0318-x (PMC6139128; doi:10.1186/s12910-018-0318-x)
Supplement: Supplementary file 2 — Table S2. Associations between the respondents’ characteristics and their desire to know each element of the ICF content. (DOCX 28 kb) [file 12910_2018_318_MOESM2_ESM.docx]

**Table S3** Associations between the respondents’ characteristics and their desire to know each element of the ICF content

|  | **B** | **SE** | **Beta** | **(95% CI)** | ***p* value** |
| --- | --- | --- | --- | --- | --- |
| 1. General items | | | | | |
| 1.1 Title of research | | | | | |
| Sex | 0.128 | 0.043 | 0.069 | (0.042 to 0.213) | 0.003 |
| Age (year) | -0.003 | 0.001 | -0.051 | (-0.006 to 0.000) | 0.033 |
| Education | 0.081 | 0.034 | 0.060 | (0.015 to 0.147) | 0.016 |
| Occupation | 0.169 | 0.069 | 0.060 | (0.033 to 0.305) | 0.015 |
| Type of research | -0.042 | 0.044 | -0.022 | (-0.128 to 0.043) | 0.331 |
| 1.2 Name of researchers | | | | | |
| Sex | 0.206 | 0.050 | 0.096 | (0.107 to 0.304) | <0.001 |
| Age (year) | -0.001 | 0.002 | -0.012 | (-0.004 to 0.002) | 0.610 |
| Education | -0.013 | 0.039 | -0.009 | (-0.090 to 0.063) | 0.731 |
| Occupation | -0.051 | 0.080 | -0.016 | (-0.209 to 0.106) | 0.523 |
| Type of research | 0.162 | 0.051 | 0.074 | (0.063 to 0.261) | 0.001 |
| 1.3 Affiliation or organization of researchers | | | | | |
| Sex | 0.138 | 0.048 | 0.068 | (0.044 to 0.233) | 0.004 |
| Age (year) | -0.002 | 0.002 | -0.033 | (-0.005 to 0.001) | 0.173 |
| Education | 0.039 | 0.037 | 0.026 | (-0.034 to 0.112) | 0.296 |
| Occupation | 0.018 | 0.077 | 0.006 | (-0.132 to 0.169) | 0.813 |
| Type of research | 0.157 | 0.048 | 0.075 | (0.062 to 0.252) | 0.001 |
| 1.4 Recognition that this is research | | | | | |
| Sex | 0.187 | 0.043 | 0.101 | (0.103 to 0.272) | <0.001 |
| Age (year) | -0.001 | 0.001 | -0.018 | (-0.004 to 0.002) | 0.454 |
| Education | 0.106 | 0.034 | 0.079 | (0.040 to 0.172) | 0.002 |
| Occupation | 0.049 | 0.069 | 0.018 | (-0.086 to 0.185) | 0.475 |
| Type of research | 0.141 | 0.044 | 0.075 | (0.056 to 0.227) | 0.001 |
| 1.5 Contact information regarding the research study | | | | | |
| Sex | 0.145 | 0.042 | 0.082 | (0.063 to 0.228) | 0.001 |
| Age (year) | -0.002 | 0.001 | -0.037 | (-0.005 to 0.001) | 0.126 |
| Education | -0.036 | 0.033 | -0.027 | (-0.100 to 0.028) | 0.276 |
| Occupation | 0.072 | 0.068 | 0.026 | (-0.061 to 0.206) | 0.288 |
| Type of research | 0.141 | 0.042 | 0.077 | (0.057 to 0.224) | 0.001 |
| 1.6 Contact information about the participant’s right | | | | | |
| Sex | 0.162 | 0.044 | 0.088 | (0.077 to 0.248) | <0.001 |
| Age (year) | -0.002 | 0.001 | -0.030 | (-0.004 to 0.001) | 0.206 |
| Education | -0.020 | 0.034 | -0.015 | (-0.086 to 0.047) | 0.557 |
| Occupation | 0.144 | 0.070 | 0.051 | (0.006 to 0.282) | 0.041 |
| Type of research | 0.189 | 0.044 | 0.100 | (0.102 to 0.276) | <0.001 |

|  | **B** | **SE** | **Beta** | **(95% CI)** | ***p* value** |
| --- | --- | --- | --- | --- | --- |
| 1.7 Source of funds and sponsors | | | | | |
| Sex | 0.190 | 0.058 | 0.077 | (0.076 to 0.304) | 0.001 |
| Age (year) | 0.004 | 0.002 | 0.054 | (0.001 to 0.008) | 0.024 |
| Education | 0.028 | 0.045 | 0.016 | (-0.060 to 0.117) | 0.531 |
| Occupation | 0.106 | 0.093 | 0.028 | (-0.075 to 0.288) | 0.250 |
| Type of research | 0.229 | 0.058 | 0.091 | (0.115 to 0.344) | <0.001 |
| 1.8 Conflict of interest | | | | | |
| Sex | 0.086 | 0.058 | 0.035 | (-0.028 to 0.201) | 0.140 |
| Age (year) | 0.005 | 0.002 | 0.072 | (0.002 to 0.009) | 0.003 |
| Education | 0.156 | 0.045 | 0.087 | (0.067 to 0.245) | 0.001 |
| Occupation | 0.394 | 0.094 | 0.104 | (0.209 to 0.579) | <0.001 |
| Type of research | 0.130 | 0.059 | 0.051 | (0.014 to 0.246) | 0.028 |
| 2. Study-specific items | | | | | |
| 2.1 Background and rationale of research | | | | | |
| Sex | 0.124 | 0.045 | 0.065 | (0.035 to 0.213) | 0.006 |
| Age (year) | -0.003 | 0.001 | -0.043 | (-0.005 to 0.000) | 0.076 |
| Education | -0.052 | 0.035 | -0.037 | (-0.120 to 0.017) | 0.142 |
| Occupation | 0.114 | 0.072 | 0.039 | (-0.028 to 0.255) | 0.116 |
| Type of research | 0.123 | 0.046 | 0.063 | (0.034 to 0.212) | 0.007 |
| 2.2 Purpose of research | | | | | |
| Sex | 0.113 | 0.040 | 0.066 | (0.034 to 0.191) | 0.005 |
| Age (year) | -0.001 | 0.001 | -0.022 | (-0.004 to 0.001) | 0.362 |
| Education | 0.008 | 0.031 | 0.006 | (-0.053 to 0.069) | 0.795 |
| Occupation | 0.205 | 0.064 | 0.079 | (0.079 to 0.330) | 0.001 |
| Type of research | 0.132 | 0.040 | 0.075 | (0.053 to 0.211) | 0.001 |
| 2.3 Eligibility of the participant | | | | | |
| Sex | 0.144 | 0.043 | 0.079 | (0.061 to 0.228) | 0.001 |
| Age (year) | -0.002 | 0.001 | -0.032 | (-0.004 to 0.001) | 0.175 |
| Education | 0.027 | 0.033 | 0.020 | (-0.038 to 0.092) | 0.417 |
| Occupation | 0.176 | 0.068 | 0.064 | (0.042 to 0.309) | 0.010 |
| Type of research | 0.121 | 0.043 | 0.065 | (0.037 to 0.205) | 0.005 |
| 2.4 Study design of research | | | | | |
| Sex | 0.209 | 0.051 | 0.097 | (0.109 to 0.308) | <0.001 |
| Age (year) | 0.001 | 0.002 | 0.013 | (-0.002 to 0.004) | 0.603 |
| Education | 0.014 | 0.039 | 0.009 | (-0.063 to 0.091) | 0.717 |
| Occupation | 0.089 | 0.081 | 0.027 | (-0.070 to 0.248) | 0.271 |
| Type of research | 0.024 | 0.051 | 0.011 | (-0.076 to 0.124) | 0.632 |
| 2.5 Interventions under investigation | | | | | |
| Sex | 0.155 | 0.040 | 0.089 | (0.075 to 0.234) | <0.001 |
| Age (year) | -0.004 | 0.001 | -0.084 | (-0.007 to -0.002) | <0.001 |
| Education | 0.017 | 0.031 | 0.013 | (-0.045 to 0.078) | 0.592 |
| Occupation | 0.214 | 0.064 | 0.081 | (0.088 to 0.340) | 0.001 |
| Type of research | 0.216 | 0.041 | 0.122 | (0.137 to 0.296) | <0.001 |
|  | **B** | **SE** | **Beta** | **(95% CI)** | ***p* value** |
| 2.6 Common adverse effects of the intervention | | | | | |
| Sex | 0.130 | 0.039 | 0.078 | (0.054 to 0.206) | 0.001 |
| Age (year) | -0.002 | 0.001 | -0.038 | (-0.004 to 0.000) | 0.107 |
| Education | 0.131 | 0.030 | 0.108 | (0.072 to 0.190) | <0.001 |
| Occupation | 0.161 | 0.062 | 0.064 | (0.040 to 0.282) | 0.009 |
| Type of research | 0.206 | 0.039 | 0.120 | (0.129 to 0.282) | <0.001 |
| 2.7 All possible adverse effects of the intervention | | | | | |
| Sex | 0.125 | 0.042 | 0.068 | (0.042 to 0.208) | 0.003 |
| Age (year) | -0.003 | 0.001 | -0.057 | (-0.006 to -0.001) | 0.015 |
| Education | 0.172 | 0.033 | 0.128 | (0.108 to 0.237) | <0.001 |
| Occupation | 0.129 | 0.068 | 0.046 | (-0.003 to 0.262) | 0.056 |
| Type of research | 0.284 | 0.043 | 0.151 | (0.200 to 0.368) | <0.001 |
| 2.8 Other options or alternative treatments | | | | | |
| Sex | 0.248 | 0.050 | 0.115 | (0.150 to 0.346) | <0.001 |
| Age (year) | -0.004 | 0.002 | -0.066 | (-0.007 to -0.001) | 0.005 |
| Education | 0.071 | 0.039 | 0.045 | (-0.005 to 0.147) | 0.066 |
| Occupation | 0.387 | 0.080 | 0.118 | (0.231 to 0.544) | <0.001 |
| Type of research | 0.231 | 0.050 | 0.104 | (0.133 to 0.330) | <0.001 |
| 2.9 Duration of the participant’s participation | | | | | |
| Sex | 0.148 | 0.045 | 0.077 | (0.060 to 0.236) | 0.001 |
| Age (year) | -0.003 | 0.001 | -0.046 | (-0.005 to 0.000) | 0.055 |
| Education | 0.047 | 0.035 | 0.034 | (-0.021 to 0.115) | 0.174 |
| Occupation | 0.243 | 0.072 | 0.083 | (0.102 to 0.384) | 0.001 |
| Type of research | 0.230 | 0.045 | 0.117 | (0.141 to 0.319) | <0.001 |
| 2.10 Schedule and procedure | | | | | |
| Sex | 0.172 | 0.041 | 0.099 | (0.092 to 0.251) | <0.001 |
| Age (year) | 0.000 | 0.001 | 0.006 | (-0.002 to 0.003) | 0.812 |
| Education | 0.023 | 0.032 | 0.018 | (-0.039 to 0.085) | 0.460 |
| Occupation | 0.158 | 0.065 | 0.060 | (0.030 to 0.285) | 0.015 |
| Type of research | 0.196 | 0.041 | 0.110 | (0.116 to 0.277) | <0.001 |
| 2.11 Identification of any experimental procedures | | | | | |
| Sex | 0.148 | 0.046 | 0.075 | (0.058 to 0.239) | 0.001 |
| Age (year) | -0.002 | 0.001 | -0.025 | (-0.004 to 0.001) | 0.294 |
| Education | 0.071 | 0.036 | 0.049 | (0.001 to 0.142) | 0.046 |
| Occupation | 0.316 | 0.074 | 0.105 | (0.171 to 0.460) | <0.001 |
| Type of research | 0.280 | 0.047 | 0.137 | (0.188 to 0.371) | <0.001 |
| 2.12 Number of participants involved | | | | | |
| Sex | 0.234 | 0.057 | 0.096 | (0.121 to 0.346) | <0.001 |
| Age (year) | -0.002 | 0.002 | -0.023 | (-0.005 to 0.002) | 0.333 |
| Education | -0.003 | 0.045 | -0.002 | (-0.091 to 0.084) | 0.940 |
| Occupation | 0.192 | 0.092 | 0.052 | (0.012 to 0.371) | 0.036 |
| Type of research | 0.074 | 0.058 | 0.030 | (-0.039 to 0.187) | 0.199 |

|  | **B** | **SE** | **Beta** | **(95% CI)** | ***p* value** |
| --- | --- | --- | --- | --- | --- |
| 2.13 Criteria for termination | | | | | |
| Sex | 0.034 | 0.045 | 0.018 | (-0.055 to 0.123) | 0.451 |
| Age (year) | -0.001 | 0.001 | -0.014 | (-0.004 to 0.002) | 0.567 |
| Education | 0.016 | 0.035 | 0.012 | (-0.053 to 0.086) | 0.641 |
| Occupation | 0.315 | 0.073 | 0.106 | (0.171 to 0.459) | <0.001 |
| Type of research | 0.267 | 0.046 | 0.134 | (0.177 to 0.357) | <0.001 |
| 3. Items related to the subject’s right | | | | | |
| 3.1 Voluntary participation | | | | | |
| Sex | 0.164 | 0.047 | 0.081 | (0.072 to 0.256) | <0.001 |
| Age (year) | -0.001 | 0.001 | -0.020 | (-0.004 to 0.002) | 0.394 |
| Education | 0.080 | 0.036 | 0.055 | (0.009 to 0.151) | 0.027 |
| Occupation | 0.353 | 0.075 | 0.115 | (0.206 to 0.499) | <0.001 |
| Type of research | 0.218 | 0.047 | 0.106 | (0.126 to 0.310) | <0.001 |
| 3.2 Consequence of withdrawal | | | | | |
| Sex | 0.105 | 0.051 | 0.048 | (0.005 to 0.206) | 0.040 |
| Age (year) | -0.003 | 0.002 | -0.042 | (-0.006 to 0.000) | 0.076 |
| Education | 0.066 | 0.040 | 0.041 | (-0.012 to 0.144) | 0.099 |
| Occupation | 0.223 | 0.083 | 0.067 | (0.061 to 0.386) | 0.007 |
| Type of research | 0.291 | 0.052 | 0.130 | (0.189 to 0.392) | <0.001 |
| 3.3 Right to receive new information | | | | | |
| Sex | 0.097 | 0.043 | 0.053 | (0.012 to 0.182) | 0.026 |
| Age (year) | -0.001 | 0.001 | -0.013 | (-0.003 to 0.002) | 0.575 |
| Education | 0.059 | 0.034 | 0.044 | (-0.007 to 0.125) | 0.081 |
| Occupation | 0.112 | 0.070 | 0.040 | (-0.026 to 0.249) | 0.111 |
| Type of research | 0.160 | 0.044 | 0.085 | (0.074 to 0.246) | <0.001 |
| 4. Items related to risk/benefit | | | | | |
| 4.1 Major foreseeable risk | | | | | |
| Sex | 0.095 | 0.041 | 0.054 | (0.015 to 0.176) | 0.020 |
| Age (year) | -0.003 | 0.001 | -0.053 | (-0.005 to 0.000) | 0.026 |
| Education | 0.089 | 0.032 | 0.069 | (0.026 to 0.151) | 0.005 |
| Occupation | 0.187 | 0.065 | 0.070 | (0.059 to 0.315) | 0.004 |
| Type of research | 0.203 | 0.041 | 0.113 | (0.122 to 0.284) | <0.001 |
| 4.2 Minor foreseeable risk | | | | | |
| Sex | 0.177 | 0.045 | 0.092 | (0.089 to 0.265) | <0.001 |
| Age (year) | -0.003 | 0.001 | -0.053 | (-0.006 to 0.000) | 0.025 |
| Education | 0.046 | 0.035 | 0.033 | (-0.022 to 0.115) | 0.184 |
| Occupation | 0.176 | 0.072 | 0.060 | (0.035 to 0.317) | 0.014 |
| Type of research | 0.287 | 0.045 | 0.145 | (0.198 to 0.376) | <0.001 |

|  | **B** | **SE** | **Beta** | **(95% CI)** | ***p* value** |
| --- | --- | --- | --- | --- | --- |
| 4.3 Possibly unforeseeable risk | | | | | |
| Sex | 0.261 | 0.049 | 0.123 | (0.164 to 0.357) | <0.001 |
| Age (year) | -0.009 | 0.002 | -0.133 | (-0.012 to -0.006) | <0.001 |
| Education | 0.066 | 0.038 | 0.043 | (-0.008 to 0.140) | 0.081 |
| Occupation | 0.232 | 0.078 | 0.072 | (0.079 to 0.386) | 0.003 |
| Type of research | 0.262 | 0.049 | 0.121 | (0.165 to 0.359) | <0.001 |
| 4.4 Direct health benefit | | | | | |
| Sex | 0.095 | 0.036 | 0.061 | (0.023 to 0.166) | 0.009 |
| Age (year) | 0.000 | 0.001 | 0.006 | (-0.002 to 0.002) | 0.816 |
| Education | -0.002 | 0.028 | -0.002 | (-0.057 to 0.053) | 0.936 |
| Occupation | 0.119 | 0.058 | 0.051 | (0.005 to 0.232) | 0.041 |
| Type of research | 0.085 | 0.037 | 0.054 | (0.013 to 0.156) | 0.021 |
| 4.5 Indirect benefit | | | | | |
| Sex | 0.149 | 0.040 | 0.088 | (0.071 to 0.228) | <0.001 |
| Age (year) | 0.001 | 0.001 | 0.026 | (-0.001 to 0.004) | 0.277 |
| Education | -0.075 | 0.031 | -0.060 | (-0.136 to -0.014) | 0.016 |
| Occupation | -0.005 | 0.064 | -0.002 | (-0.131 to 0.120) | 0.933 |
| Type of research | 0.063 | 0.040 | 0.036 | (-0.016 to 0.142) | 0.117 |
| 4.6 Societal benefit | | | | | |
| Sex | 0.060 | 0.043 | 0.034 | (-0.024 to 0.143) | 0.159 |
| Age (year) | -0.001 | 0.001 | -0.015 | (-0.003 to 0.002) | 0.535 |
| Education | -0.057 | 0.033 | -0.044 | (-0.122 to 0.007) | 0.083 |
| Occupation | 0.031 | 0.069 | 0.011 | (-0.104 to 0.166) | 0.652 |
| Type of research | 0.049 | 0.043 | 0.027 | (-0.035 to 0.133) | 0.256 |
| 4.7 Post-trial benefit or provision | | | | | |
| Sex | 0.118 | 0.044 | 0.064 | (0.032 to 0.205) | 0.007 |
| Age (year) | 0.001 | 0.001 | 0.016 | (-0.002 to 0.004) | 0.506 |
| Education | 0.015 | 0.034 | 0.011 | (-0.052 to 0.081) | 0.668 |
| Occupation | 0.110 | 0.071 | 0.039 | (-0.029 to 0.249) | 0.121 |
| Type of research | 0.055 | 0.044 | 0.029 | (-0.032 to 0.142) | 0.212 |
| 5. Items related to data and sample storage | | | | | |
| 5.1 Confidentiality and the limit of confidentiality | | | | | |
| Sex | 0.119 | 0.046 | 0.060 | (0.028 to 0.210) | 0.011 |
| Age (year) | -0.003 | 0.001 | -0.055 | (-0.006 to 0.000) | 0.022 |
| Education | 0.113 | 0.036 | 0.079 | (0.043 to 0.184) | 0.002 |
| Occupation | 0.193 | 0.075 | 0.064 | (0.046 to 0.340) | 0.010 |
| Type of research | 0.095 | 0.047 | 0.047 | (0.003 to 0.187) | 0.043 |
| 5.2 Storage of human material | | | | | |
| Sex | 0.150 | 0.054 | 0.065 | (0.043 to 0.257) | 0.006 |
| Age (year) | -0.001 | 0.002 | -0.013 | (-0.004 to 0.002) | 0.596 |
| Education | 0.012 | 0.042 | 0.007 | (-0.071 to 0.095) | 0.782 |
| Occupation | 0.242 | 0.088 | 0.068 | (0.069 to 0.415) | 0.006 |
| Type of research | 0.179 | 0.055 | 0.076 | (0.071 to 0.287) | 0.001 |
|  | **B** | **SE** | **Beta** | **(95% CI)** | ***p* value** |
| 5.3 Reuse of human material | | | | | |
| Sex | 0.179 | 0.053 | 0.080 | (0.076 to 0.283) | 0.001 |
| Age (year) | -0.005 | 0.002 | -0.069 | (-0.008 to -0.002) | 0.004 |
| Education | 0.086 | 0.041 | 0.053 | (0.005 to 0.166) | 0.036 |
| Occupation | 0.148 | 0.085 | 0.043 | (-0.019 to 0.314) | 0.083 |
| Type of research | 0.091 | 0.053 | 0.039 | (-0.014 to 0.195) | 0.088 |
| 6. Items related to monetary issues | | | | | |
| 6.1 Payment and/or remuneration | | | | | |
| Sex | 0.166 | 0.055 | 0.072 | (0.059 to 0.273) | 0.002 |
| Age (year) | 0.001 | 0.002 | 0.015 | (-0.002 to 0.004) | 0.537 |
| Education | -0.011 | 0.042 | -0.006 | (-0.094 to 0.072) | 0.800 |
| Occupation | 0.227 | 0.088 | 0.064 | (0.053 to 0.400) | 0.010 |
| Type of research | 0.168 | 0.055 | 0.071 | (0.059 to 0.276) | 0.002 |
| 6.2 Anticipated expense | | | | | |
| Sex | 0.135 | 0.052 | 0.061 | (0.032 to 0.237) | 0.010 |
| Age (year) | 0.001 | 0.002 | 0.009 | (-0.003 to 0.004) | 0.694 |
| Education | 0.089 | 0.041 | 0.055 | (0.009 to 0.168) | 0.029 |
| Occupation | 0.287 | 0.084 | 0.085 | (0.122 to 0.451) | 0.001 |
| Type of research | 0.210 | 0.053 | 0.093 | (0.107 to 0.313) | <0.001 |
| 6.3 Compensation for injury | | | | | |
| Sex | 0.165 | 0.043 | 0.091 | (0.081 to 0.249) | <0.001 |
| Age (year) | -0.001 | 0.001 | -0.024 | (-0.004 to 0.001) | 0.324 |
| Education | 0.037 | 0.033 | 0.028 | (-0.028 to 0.103) | 0.260 |
| Occupation | 0.184 | 0.069 | 0.066 | (0.048 to 0.321) | 0.008 |
| Type of research | 0.163 | 0.043 | 0.087 | (0.078 to 0.248) | <0.001 |
