# Supplementary material for: What information and the extent of information research participants need in informed consent forms: a multi-country survey
Source: BMC Med Ethics. 2018 Sep 15;19:79. doi: 10.1186/s12910-018-0318-x (PMC6139128; doi:10.1186/s12910-018-0318-x)
Supplement: Supplementary file 3 — Table S3. The maximum, acceptable number of pages in the informed consent form and its comparisons among countries. (DOCX 16 kb) [file 12910_2018_318_MOESM3_ESM.docx]

**Table S4** The maximum, acceptable number of pages in the informed consent form and its comparisons among countries

| **Mean (±SD) of the maximum, acceptable number of pages** | **India**  4.96 (±3.17) | **Indonesia**  4.10 (±3.32) | **Malaysia**  6.98 (±5.66) | **Philippines**  7.68 (±6.54) | **Sri Lanka**  3.71 (±3.27) | **Taiwan**  12.25 (±10.76) | **Thailand**  5.32 (±3.06) |
| --- | --- | --- | --- | --- | --- | --- | --- |
| **India**  4.96 (±3.17) |  | 0.86  (-0.49 to 2.22) | -2.02  (-3.17 to -0.86) | -2.72  (-4.15 to -1.28) | 1.25  (-0.16 to 2.66) | -7.29  (-8.80 to -5.78) | -0.36  (-3.96 to 3.25) |
| **Indonesia**  4.10 (±3.32) | -0.86  (-2.22 to 0.49) |  | -2.88  (-4.20 to -1.56) | -3.58  (-5.15 to -2.01) | 0.39  (-1.16 to 1.93) | -8.15  (-9.79 to -6.52) | -1.22  (-4.88 to 2.44) |
| **Malaysia**  6.98 (±5.66) | 2.02  (0.86 to 3.17) | 2.88  (1.56 to 4.20) |  | -0.70  (-2.10 to 0.70) | 3.27  (1.89 to 4.64) | -5.27  (-6.75 to -3.80) | 1.66  (-1.93 to 5.25) |
| **Philippines**  7.68 (±6.54) | 2.72  (1.28 to 4.15) | 3.58  (2.01 to 5.15) | 0.70  (-0.70 to 2.10) |  | 3.97  (2.35 to 5.58) | -4.57  (-6.28 to -2.87) | 2.36  (-1.33 to 6.05) |
| **Sri Lanka**  3.71 (±3.27) | -1.25  (-2.66 to 0.16) | -0.39  (-1.93 to 1.16) | -3.27  (-4.64 to -1.89) | -3.97  (-5.58 to -2.35) |  | -8.54  (-10.22 to -6.86) | -1.61  (-5.28 to 2.08) |
| **Taiwan**  12.25 (±10.76) | 7.29  (5.78 to 8.80) | 8.15  (6.52 to 9.79) | 5.27  (3.80 to 6.75) | 4.57  (2.87 to 6.28) | 8.54  (6.86 to 10.22) |  | 6.94  (3.22 to 10.65) |
| **Thailand**  5.32 (±3.06) | 0.36  (-3.25 to 3.96) | 1.22  (-2.44 to 4.88) | -1.66  (-5.25 to 1.93) | -2.36  (-6.05 to 1.33) | 1.61  (-2.08 to 5.28) | -6.94  (-10.65 to -3.22) |  |

The mean score difference is in the cell in common between the row-defining country and the column-defining country. Underlined entries indicate statistical significance (*p* value <0.05) by one-way ANOVA, followed by Tukey *post hoc* test.
