# Supplementary figures and images for: What information and the extent of information research participants need in informed consent forms: a multi-country survey
Source: BMC Med Ethics. 2018 Sep 15;19:79. doi: 10.1186/s12910-018-0318-x (PMC6139128; doi:10.1186/s12910-018-0318-x)

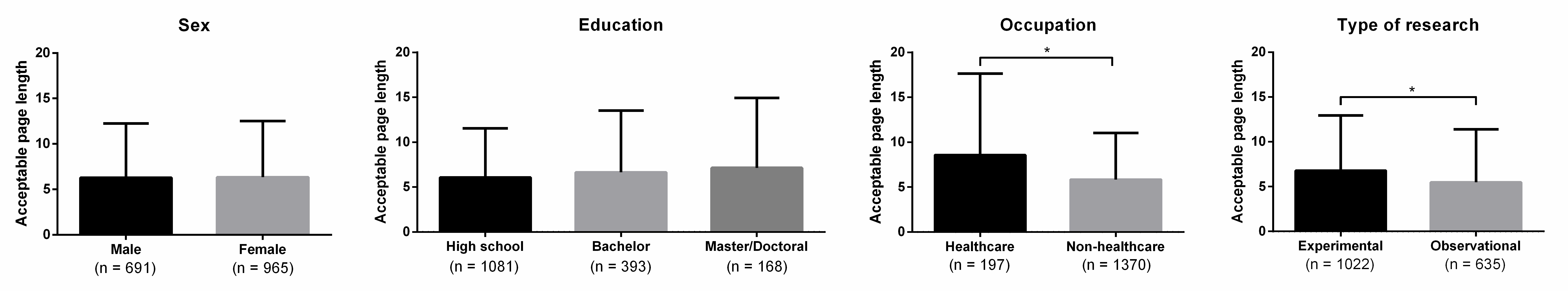

Supplement: Supplementary file 4 — Figure S1. Differences in the acceptable page length among respondents with different genders, educational levels, occupations, and types of research involved. (TIF 512 kb) [file 12910_2018_318_MOESM4_ESM.tif]
